# Hypnotizability and Catechol-O-Methyltransferase (COMT) polymorphysms in *Italians*

**DOI:** 10.3389/fnhum.2013.00929

**Published:** 2014-01-06

**Authors:** Silvano Presciuttini, Alessandro Gialluisi, Serena Barbuti, Michele Curcio, Fabrizio Scatena, Giancarlo Carli, Enrica L. Santarcangelo

**Affiliations:** ^1^Laboratory of Cognitive and Behavioral Neurosciences, Department of Translational Research and New Technologies in Medicine and Surgery, University of PisaPisa, Italy; ^2^Language and Genetics Department, Max Planck Institute for PsycholinguisticsNijmegen, Netherlands; ^3^Immunohematology Unit, Azienda Ospedaliera–Universitaria PisanaPisa, Italy; ^4^Department of Medicine, Surgery and Neuroscience, University of SienaSiena, Italy

**Keywords:** hypnotizability, attention, COMT, absorption, selective genotyping, haplotype analysis

## Abstract

Higher brain dopamine content depending on lower activity of Catechol-*O*-Methyltransferase (COMT) in subjects with high hypnotizability scores (*highs*) has been considered responsible for their attentional characteristics. However, the results of the previous genetic studies on association between hypnotizability and the COMT single nucleotide polymorphism (SNP) rs4680 (Val^158^Met) were inconsistent. Here, we used a selective genotyping approach to re-evaluate the association between hypnotizability and *COMT* in the context of a two-SNP haplotype analysis, considering not only the Val^158^Met polymorphism, but also the closely located rs4818 SNP. An Italian sample of 53 *highs*, 49 low hypnotizable subjects (*lows*), and 57 controls, were genotyped for a segment of 805 bp of the *COMT* gene, including Val^158^Met and the closely located rs4818 SNP. Our selective genotyping approach had 97.1% power to detect the previously reported strongest association at the significance level of 5%. We found no evidence of association at the SNP, haplotype, and diplotype levels. Thus, our results challenge the dopamine-based theory of hypnosis and indirectly support recent neuropsychological and neurophysiological findings reporting the lack of any association between hypnotizability and focused attention abilities.

## INTRODUCTION

The cognitive trait of hypnotizability (Green et al., 2005) – the ability to accept hypnotic suggestions – has been classically attributed to peculiar characteristics of the supervisory attentional system (Norman and Shallice, 1986; Posner and Fan, 2004) allowing a more flexible attentional control in the subjects scoring high (*highs*) at hypnotizability scales. In fact, a few neuropsychological (Tellegen and Atkinson, 1974; Zachariae et al., 2000) and genetic studies (Lichtenberg et al., 2000; Raz, 2005; Raz et al., 2006; Szekely et al., 2010) have suggested greater abilities of focused attention in *highs* with respect to low hypnotizable individuals (*lows*), based on higher dopaminergic activity.

In the general population, attention seems to be more efficiently controlled in subjects with the Met/Met or Val/Met variant of the single nucleotide polymorphism (SNP) rs4680 at the catechol-*O*-methiltransferase (COMT) gene than in the homozygous Val/Val individuals (Seamans and Yang, 2004). In fact, the Met/Met variant shows 40% less enzymatic activity than the Val/Val and, thus, is associated with higher dopamine levels in the prefrontal (Roussos et al., 2008) and anterior cingulate cortex (Blasi et al., 2005).

The three association studies conducted so far on the relation between the COMT Val^158^Met polymorphism and hypnotizability have provided inconsistent results. Two of them (Lichtenberg et al., 2000; Raz, 2005) applied analysis of variance on the hypnotizability scores in subjects stratified by the COMT genotype. In this approach, a sample of subjects not selected for hypnotizability (thus representing the distribution of this trait in the general population) is genotyped, and ANOVA is used to test the differences of the mean hypnotizability scores among the genotypes. Both studies reported a higher mean score of hypnotizability in heterozygotes (Met/Val) than in both homozygotes (Val/Val, Met/Met), but in one of them the association between hypnotizability and COMT polymorphism was significant in females only (Lichtenberg et al., 2000). On the contrary, the third study (Szekely et al., 2010) using the same approach reported intermediate hypnotizability scores in heterozygotes; these authors also contrasted the *highs* and *lows* recruited in the sample for genotype frequencies, and found a significantly higher frequency of the Val allele among *highs*.

Thus, the first aim of the present study was to re-evaluate the relationship between the rs4680 (Val^158^Met) COMT variant and hypnotizability through a selective genotyping approach.

It should be noticed also that the COMT locus is polymorphic for many other SNPs that may interact in a complex way to determine phenotypic differences among individuals (Diatchenko et al., 2005; Nackley et al., 2006; Roussos et al., 2008). This occurs, for instance, for the coding regions rs4633 (C/T, synonymous), rs4818 (C/G, synonymous), and rs4680 (G/A, Val^158^Met), which are strongly associated with experimental pain sensitivity (Diatchenko et al., 2005). Thus, the second aim of the present study was to conduct the analysis, for the first time on hypnotisability, at the haplotype level, including the two closely located SNPs rs4818 (Leu^136^Leu) and rs4680 (Val^158^Met).

## MATERIALS AND METHODS

### SUBJECTS

After signing an informed consent describing the nature and procedure of the study, 102 unpaid healthy subjects volunteered for the study which was approved by the Ethical Committee of the University of Pisa. Hypnotizability was evaluated according to the *Italian* version (Weitzenhoffer and Hilgard, 1959; De Pascalis et al., 2000) of the Stanford Hypnotic Susceptibility Scale (SHSS, form A). The participants were 53 *highs* (19 M, 34 F; SHSS mean score: 10.26 + 1.04) and 49 *lows* (22 M, 27 F; SHSS mean score: 0.33 + 0.59) selected from a database including 1043 students of the Universities of Pisa and Siena (410 M, 633 F). As a control group representative of the general population, 57 umbilical cords (Controls) from the Immuno-hemathology Unit Bank at the Azienda Ospedaliera–Universitaria Pisana, were genotyped anonymously (Controls). Consensus on the employment of the umbilical cords for research had been obtained from mothers at the Azienda Ospedaliera–Universitaria Pisana soon after delivery.

### DNA EXTRACTION, AMPLIFICATION AND ANALYSIS

Genomic DNA was isolated by the QIAamp DNA Blood kit (QIAGEN GmbH, Hilden, Germany) according to manufacture’s instructions from *highs*’ and *lows’* peripheral blood leukocytes. The same was done with umbilical cords samples from (Controls). For privacy requirements, blood samples were coded anonymously. The DNA extracted from 200 μl of blood was diluted with 200 μl of H2O, quantified by UV measurement at OD 260 nm and stored at -20°C until further processing. Later, the DNA sample was restored at a normal temperature and underwent a polymerase chain reaction (PCR) aimed at amplifying the target region in the COMT gene, i.e., a portion of 805 bp containing the exon 4, in which the SNP rs4680 resides. The amplification (performed on a PTC 100 Thermal Cycler, MJ Research, Watertown, MA, USA) was done in 50 μl reactions containing 5 μl of 10X Buffer solution, 0.2 μM of each primer (COMT-F: 5′-ATCCAAGTTCCCCTCTCTCCACCTG-3′; COMT-R: 5′-GTTGGGGCTCACCTCCAAGAGAAGC-3′), 0.2 mM deoxynucleoside triphosphates (dNTPs), 1.5 mM of MgCl2, 2.5 U Taq DNA Polymerase, Recombinant (Invitrogen by life technologies), and ~100 ng of genomic DNA, along with H2O to complete the total reaction volume. PCR conditions consisted of an initial denaturation step at 96°C for 2 min followed by 35 cycles on a thermocycler (denaturation at 96°C for 30 s, annealing at 68°C for 20 s, and extension at 72°C for 60 s). After specific amplification the PCR fragments were purified using QIAquick PCR Purification microcentrifuge columns. Sequencing reactions were carried out using forward and reverse primers (COMT-F: 5′-ATCCAAGTTCCCCTCTCTCCACCTG-3′ and COMT seq, R, 5′-CCTTTTTCCAGGTCTGACAA-3′) and BigDye Terminator v3.1 in accord to protocol (Applied Biosystems, USA). The sequencing were run on the ABI 3130xl Prism Genetic Analyzer (Applied Biosystems) and analyzed using the software SeqScape v.2.5 (Applied Biosystems).

A DNA fragment of 240 bp of the COMT gene containing the well-known non-synonymous SNP rs4680 (G/A at position 472, or Val^158^Met) was sequenced in *highs, lows,* and Controls. In addition to rs4680, a second SNP was identified at position 408, namely, the synonymous rs4818 (C/G, or Leu^136^Leu).

### STATISTICAL ANALYSIS

Adherence of genotype frequencies to Hardy–Weinberg equilibrium was assessed by goodness-of-fit tests. Heterogeneity of allele frequencies among population samples was assessed by contingency-table χ^2^ analysis. Difference of allele frequency between *highs* and *lows* was measured by calculating odds ratio and 95% confidence limits. Maximum likelihood estimates of two-locus haplotype frequencies were obtained by the expectation-maximization (EM) algorithm (Terwilliger and Ott, 1994). Power analysis was evaluated by the arcsine transformation of Cohen (Cohen, 1988; Motulsky, 2010). All calculations were performed in Excel.

## RESULTS

**Table 1** shows the genotypes at the COMT Val^158^Met polymorphism and the *Met* allele frequency in *highs*, *lows,* and Controls; no heterogeneity of allele frequency was detected (χ^2^ = 4.27, d.f. = 2, *p* = 0.118). The odds ratio of the Val allele for *highs* and *lows* (2 × 2 table) was 0.7; 95% Confidence Interval: 0.4–1.3.

**Table 1:** Genotypes at COMT Val^**158**^Met polymorphism in highs, lows, and Controls.

Sample type	MetMet	MetVal	ValVal	Total	p(A)^1^
	AA	AG	GG
*highs*	11	25	17	53	0.443
%	*20.8*	*47.2*	*32.1*		
*lows*	10	16	23	49	0.367
%	*20.4*	*32.7*	*46.9*		
Controls	12	34	11	57	0.569

1 Metallele frequency.

**Table 2** shows the genotype counts of rs4818 (C/G, or Leu^136^Leu) and rs4680 (G/A, or Val^158^Met) in the form of two-SNP genotypes, for each of our three samples. Hardy–Weinberg equilibrium tests were performed for each of the two SNPs, separately for males and females. No significant deviation was detected in any of these subsamples.

**Table 2:** Joint genotype distribution of rs4818 (C/G, or Leu^136^Leu) and rs4680 (G/A, or Val^158>^Met), in three population samples.

*highs*						*lows*						Controls					
	rs4680^1^					rs4680^1^					rs4680^1^			
rs4818	AA	GA	GG	Total	rs4818	AA	GA	GG	Total	rs4818	AA	GA	GG	Total
CC	11	8	3	22	CC	10	4	2	16	CC	11	12	2	25
CG	0	17	4	21	CG	0	12	7	19	CG	1	21	2	24
GG	0	0	10	10	GG	0	0	14	14	GG	0	1	7	8
Total	11	25	17	53	Total	10	16	23	49	Total	12	34	11	57

1 AA, (MetMet); GA, (MetVal); GG, (ValVal).

**FIGURE 1:**
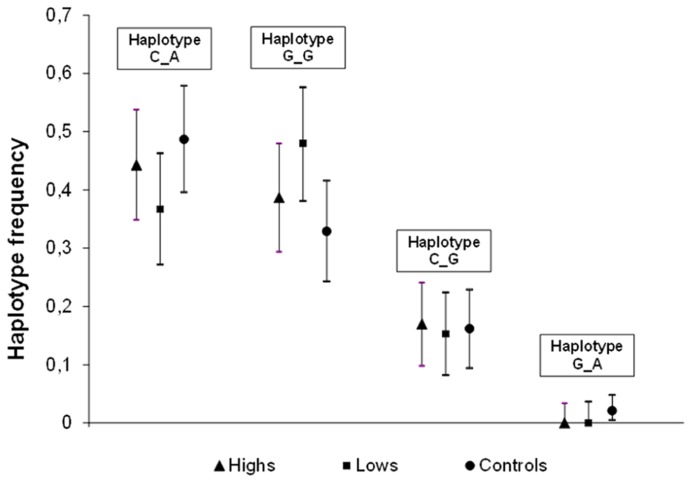
**Estimated haplotype frequencies of the two SNPs, rs4818 (C/G) and rs4680 (G/A, Val^158^Met) in *highs* (*N* = 53), *lows* (*N* = 49), Controls (*N* = 57)**. Marker order: rs4818–rs4680.

The EM algorithm produced the haplotype frequency estimates shown in **Figure 1**. One of the four possible haplotypes (G_A, in the order rs4818–rs4680) was absent from both *highs* and *lows*, meaning complete linkage disequilibrium, whereas it was present at low frequency (0.02) in the control sample. The large overlap of the 95% confidence intervals of the three samples makes it clear that there is no association between hypnotizability and these COMT haplotypes. Indeed, there was no evidence of heterogeneity (χ^2^ = 3.78, d.f. = 5, *p* = 0.582) in *highs* and *lows* also for the absolute frequencies of the two-SNP diplotypes.

## DISCUSSION

The present study does not show any association between COMT polymorphisms and hypnotizability at the SNP, haplotype, and diplotype levels.

### GENETIC FINDINGS AND NEUROPSYCHOLOGICAL EVIDENCE ON HYPNOTIZABILITY-RELATED ATTENTIONAL ABILITIES

Previous studies (Lichtenberg et al., 2000; Raz, 2005; Raz et al., 2006; Szekely et al., 2010) presented some evidence of association between the Val^158^Met polymorphism and hypnotisability (**Table 3**).

**Table 3:** Genetic association studies between the COMT Val^158^Met polymorphism and hypnotizability.

	Sample type	MetMet	MetVal	ValVal	Total	p(A)^1^	Remarks
		AA	AG	GG
***ANOVA based approaches***
Lichtenberg etal. (2000)	Unstratified	77	41	19	137	0.712	Association significant in females only; highest hypnotizability score in heterozygotes
	*%*	*56.2*	*29.9*	*13.9*			
	Mean HS^2^	**5.2**	**6.6**	**4.5**			
Raz (2005)	Unstratified	18	33	25	76	0.454	Highest hypnotizability score in heterozygotes; no significance test provided
	%	*23.7*	*43.4*	*32.9*			
	Mean HS	**6.1**	**7.6**	**5.9**			
Szekely etal. (2010)	Unstratified	30	66	31	127	0.496	ANOVA significant for genotype effect (*p* = 0.016); medium score in heterozygotes
	%	*23.6*	*52.0*	*24.4*			
	Mean HS	**4.1**	**4.7**	**5.9**			
***Categorical data analysis***
Szekely etal. (2010)	*highs* (mean HS 9.3 ± 1.0)	1	9	9	19	0.289	χ^2^significant for heterogeneity (*p* = 0.009); Odds Ratio for the Val allele (2 × 2 table) = 3.0; 95% CI^3^: 1.4–6.7 (calculated from published data)^4^
	%	*5.2*	*47.4*	*47.4*			
	*lows* (mean HS 2.6 ± 0.7)	15	34	9	58	0.552	
	%	*25.9*	*58.6*	*15.5*			

*^1^Metallele frequency; ^2^Hypnotizability Score; ^3^Confidence Interval; ^4^The corresponding value of our data was 0.7; 95% CI: 0.4–1.3.
*

The most important discrepancy concerns our results and those reported by Szekely et al. (2010). That work found a significant difference in allele frequencies between *highs* and *lows,* but its power was limited by the necessarily small proportion of *highs* in the samples (*N* = 19), which is due to the distribution of hypnotizability scores in the general population (Balthazard and Woody, 1989; De Pascalis et al., 2000; Carvalho et al., 2008). Our alternative approach of selective genotyping, in which individuals are sampled from the opposite tails of a quantitative trait, can substantially increase the power of population-based associations studies (Schork et al., 2000; Van Gestel et al., 2000).

It should be noted that the odds ratio of the Val allele (recalculated from published data) is 3.0 in the work by Szekely and coll. (Szekely et al., 2010), whereas it is 0.7 in our data, and the 95% CI do not overlap. The power of our study to detect significant heterogeneity of allele frequency between *highs* and *lows*, if their frequency were as in (Szekely et al., 2010), was 97.1% at the significance level of 5%, and it was 89.8% at the significance level of 1%.

Theoretically, the different methods of hypnotic assessment between studies might account for the different results, but we consider this unlikely, as the methods used in the present and in other works provide highly correlated results (Sheehan and McConkey, 1982). Another factor possibly accounting for the discrepancy is a different level of association in different populations; this can happen if the association is caused by a nearby locus that shows variable levels of linkage disequilibrium among populations.

The present results are in line with the findings showing the absence of any hypnotizability-related difference in attentional tests (Varga et al., 2011), and also the absence of significant correlation between COMT polymorphism and executive attention performance as measured by Posner Attentional Network Test (Fossella et al., 2002). Moreover, recent neuropsychological studies contrast the classical view of hypnotizability based on high abilities of focused attention and attribute the hypnotizability-related cognitive characteristics to impaired frontal executive functions inducing a lower capacity to disengage attention from its current focus (Jamieson and Sheehan, 2004; Egner et al., 2005). Finally, the assumption that the larger content of the homovanillic acid (HA) found in *highs* (Spiegel and King, 1992) depends on reduced DOPA catabolism (responsible for high abilities of focused attention) is weak, as HA is a catabolite of both dopamine and norepinephrine and its content in the cerebrospinal fluid derives from their catabolism in several neural circuits (Gu, 2002) not necessarily including those responsible for focused attention.

### MECHANISMS INDEPENDENT OF COMT POLYMORPHISMS POTENTIALLY INVOLVED IN HYPNOTIZABILITY-RELATED ATTENTIONAL CHARACTERISTICS

The *highs*’ attention seems to be stable rather than flexible. A few authors suggest that the carriers of the Met allele might be comparatively high in cognitive stability, but low in cognitive flexibility (Cools, 2008; Cools and D’Esposito, 2009; Colzato et al., 2010). High flexibility would be associated with great distractibility, while high stability may be related to scarce distractibility (Goschke, 2000), as suggested for *highs* (Tellegen and Atkinson, 1974; Lichtenberg et al., 2000,^2004^; Zachariae et al., 2000; Raz, 2005; Raz et al., 2006). The balance between cognitive flexibility and stability (Cools and D’Esposito, 2009; Darvas and Palmiter, 2011) could depend on the interaction between the dopaminergic circuits of the prefrontal cortex (where the catecholamines metabolism relies mainly on the activity of the COMT) and of the striatum, where the catecholamines metabolism depends mostly on the mono amino-oxidase (MAO) enzymatic system (Darvas and Palmiter, 2011). Actually, polymorphisms in MAO have also been found associated with executive attention and with alerting efficiency (Fossella et al., 2002).Thus, different attentional performance could be accounted for by a peculiar balance between the catecholamines degradation occurring in different brain structures.

However, the existence of multiple subtypes of *highs* and *lows* (Balthazard and Woody, 1989; Pekala and Forbes, 1997; Green and Lynn, 2011; Terhune et al., 2011) suggests that it is unlikely that one biological determinant may account for such a complex trait like the susceptibility to hypnosis, and we may expect that several neurotransmitters and neuromodulators influence hypnotizability (Ott et al., 2005; Klinkenberg et al., 2011). Recent evidence suggests a role for nitric oxide (NO) because the hypnotizability-related vascular responses to cognitive and physical stimulation indicate greater NO availability in the *highs’* vessels (Jambrik et al., 2004; Jambrik et al., 2005). In the brain, endothelial NO is responsible for basal vascular tone, interacts with other mediators in its modulation, and acts as a neurotransmitter after diffusion to the extracellular compartment (Andresen et al., 2006). Using an *in vivo* brain microdialysis technique, it has been demonstrated that NO significantly increases the release of acetylcholine and decreases the release of dopamine in the rat striatum (Guevara-Guzman et al., 1994), while increasing its metabolism (Nabeshima et al., 1987; Löscher et al., 1991). Thus, a greater NO availability modulating both dopamine and acetylcholine production may account for the observed higher HA content in the cerebrospinal fluid (Spiegel and King, 1992), higher arousal (Castellani and Sebastiani, 2008) and greater attentional stability (Colzato et al., 2010) of *highs* with respect to *lows*.

## CONCLUSION

The observed absence of any association between hypnotizability and COMT polymorphisms/haplotypes prompts reconsideration of the theory indicating a generally reduced brain DOPA catabolism as responsible for the attentional abilities of the subjects with high hypnotizability. The findings on the nitric oxide vascular availability open frontier research on possible alternative bases.

## AUTHOR CONTRIBUTIONS

Silvano Presciuttini, Giancarlo Carli, and Enrica L. Santarcangelo have designed the study and written the paper; Serena Barbuti, Michele Curcio, and Fabrizio Scatena have performed the DNA analysis; Alessandro Gialluisi and Silvano Presciuttini have done statistical analyses.

## Conflict of Interest Statement

The authors declare that the research was conducted in the absence of any commercial or financial relationships that could be construed as a potential conflict of interest.
